# Supplementary material for: A non-canonical mechanism of GPCR activation
Source: Nat Commun. 2024 Nov 16;15:9938. doi: 10.1038/s41467-024-54103-6 (PMC11569127; doi:10.1038/s41467-024-54103-6)
Supplement: Supplementary file 1 — Supplementary Information [file 41467_2024_54103_MOESM1_ESM.pdf]

Supplementary Information for:

A non-canonical mechanism of GPCR activation

Alexander S. Powers[1,2,3,4,5]*, Aasma Khan[6]*, Joseph M. Paggi[2,3,4,5], Naomi R. Latorraca[2,3,4,5,7,11], Sarah Souza[6], Jerry Di Salvo[8], Jun Lu[9,12], Stephen M. Soisson[9,13], Jennifer M. Johnston[10], Adam B. Weinglass[6], Ron O. Dror[2,3,4,5,7]

[1] Department of Chemistry, Stanford University, Stanford, CA 94305, USA
[2] Department of Computer Science, Stanford University, Stanford, CA 94305, USA
[3] Department of Molecular and Cellular Physiology, Stanford University School of Medicine, Stanford, CA 94305, USA
[4] Department of Structural Biology, Stanford University School of Medicine, Stanford, CA 94305, USA
[5] Institute for Computational and Mathematical Engineering, Stanford University, Stanford, CA 94305, USA
[6] Department of Quantitative Biology, Merck & Co., Inc., West Point, PA, USA
[7] Biophysics Program, Stanford University, Stanford, CA 94305, USA
[8] Evotec, Princeton, NJ 08540, USA
[9] Department of Structural Chemistry, Merck & Co., Inc., West Point, PA, USA
[10] Department of Modeling and Informatics, Merck & Co., Inc., Rahway, NJ, USA

Present addresses:
[11] Department of Biochemistry and Molecular Biophysics, Columbia University Irving Medical Center, New York, NY
[12] Small Molecule Discovery, Zai Lab (US) LLC, 314 Main Street, Suite 04-100, Cambridge, MA 02142
[13] Protein Therapeutics and Structural Biology, Odyssey Therapeutics, 51 Sleeper Street, Suite 800, Boston, MA 02210

*These authors contributed equally to this work.
Correspondence to: ron.dror@stanford.edu

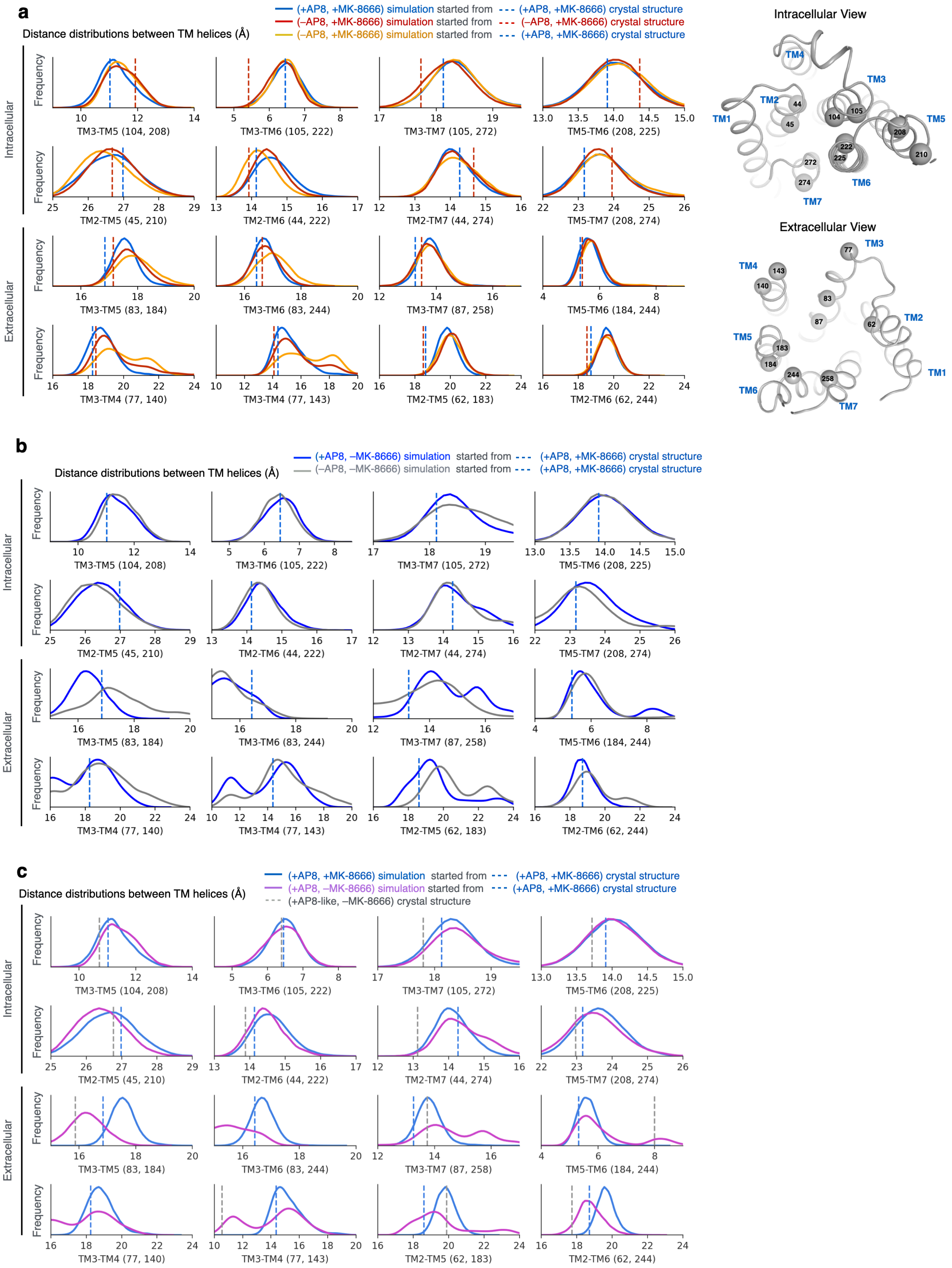
a
Distance distributions between TM helices (Å)
(+AP8, +MK-8666) simulation started from (+AP8, +MK-8666) crystal structure
(–AP8, +MK-8666) simulation started from (–AP8, +MK-8666) crystal structure
(–AP8, +MK-8666) simulation started from (+AP8, +MK-8666) crystal structure
Intracellular
Extracellular
Frequency
TM3-TM5 (104, 208)
TM3-TM6 (105, 222)
TM3-TM7 (105, 272)
TM5-TM6 (208, 225)
TM2-TM5 (45, 210)
TM2-TM6 (44, 222)
TM2-TM7 (44, 274)
TM5-TM7 (208, 274)
TM3-TM5 (83, 184)
TM3-TM6 (83, 244)
TM3-TM7 (87, 258)
TM5-TM6 (184, 244)
TM3-TM4 (77, 140)
TM3-TM4 (77, 143)
TM2-TM5 (62, 183)
TM2-TM6 (62, 244)
Intracellular View
Extracellular View
TM1
TM2
TM3
TM4
TM5
TM6
TM7
b
Distance distributions between TM helices (Å)
(+AP8, –MK-8666) simulation started from (+AP8, +MK-8666) crystal structure
(–AP8, –MK-8666) simulation started from (+AP8, +MK-8666) crystal structure
c
Distance distributions between TM helices (Å)
(+AP8, +MK-8666) simulation started from (+AP8, +MK-8666) crystal structure
(+AP8, –MK-8666) simulation started from (+AP8, +MK-8666) crystal structure
(+AP8-like, –MK-8666) crystal structure

Supplementary Figure 1. AP8 has minimal effect on the conformation of key transmembrane helices in simulation. (a) Distributions of distances describing conformation of transmembrane helices under different simulation conditions: blue started from AP8-bound crystal structure (PDB 5TZY), red started from AP8-absent crystal structure (PDB 5TZR), and orange started from AP8-bound crystal structure with AP8 removed. Distances between transmembrane helices were measured using indicated pairs of Cα atoms on the intracellular or extracellular end of each helix. Dotted vertical lines show distances in the initial crystal structures. Distributions calculated using simulation frames after 500 ns from 10 independent simulations for each condition. Images at left show the residues used to calculate these distances, with spheres representing Cα atoms in the AP8-absent crystal structure (grey cartoon). **(b)** As an additional control, we applied the same analysis to simulations *without* partial agonist MK-8666, to test the effect of AP8 in the absence of the orthosteric ligand. Grey distributions are simulations started from the AP8-bound crystal structure (PDB 5TZY) with both MK-8666 and AP8 removed, blue distributions are started from the same crystal structure with only MK-8666 removed. Means for each metric, averaged from N=5 independent simulations for each condition, were not statistically different between the conditions (two-sided student *t*-test). **(c)** As a positive control, we applied the same analysis to simulations with and without FFAR1 partial agonist MK-8666 (starting from PDB 5TZY), which binds closer to "canonical" orthosteric site within the helical bundle. MK-8666 does have a substantial effect on key transmembrane helices which shifted by 2-6 angstroms. Dotted grey lines indicate distances in the MK-8666 free crystal structure (PDB 5KW2).

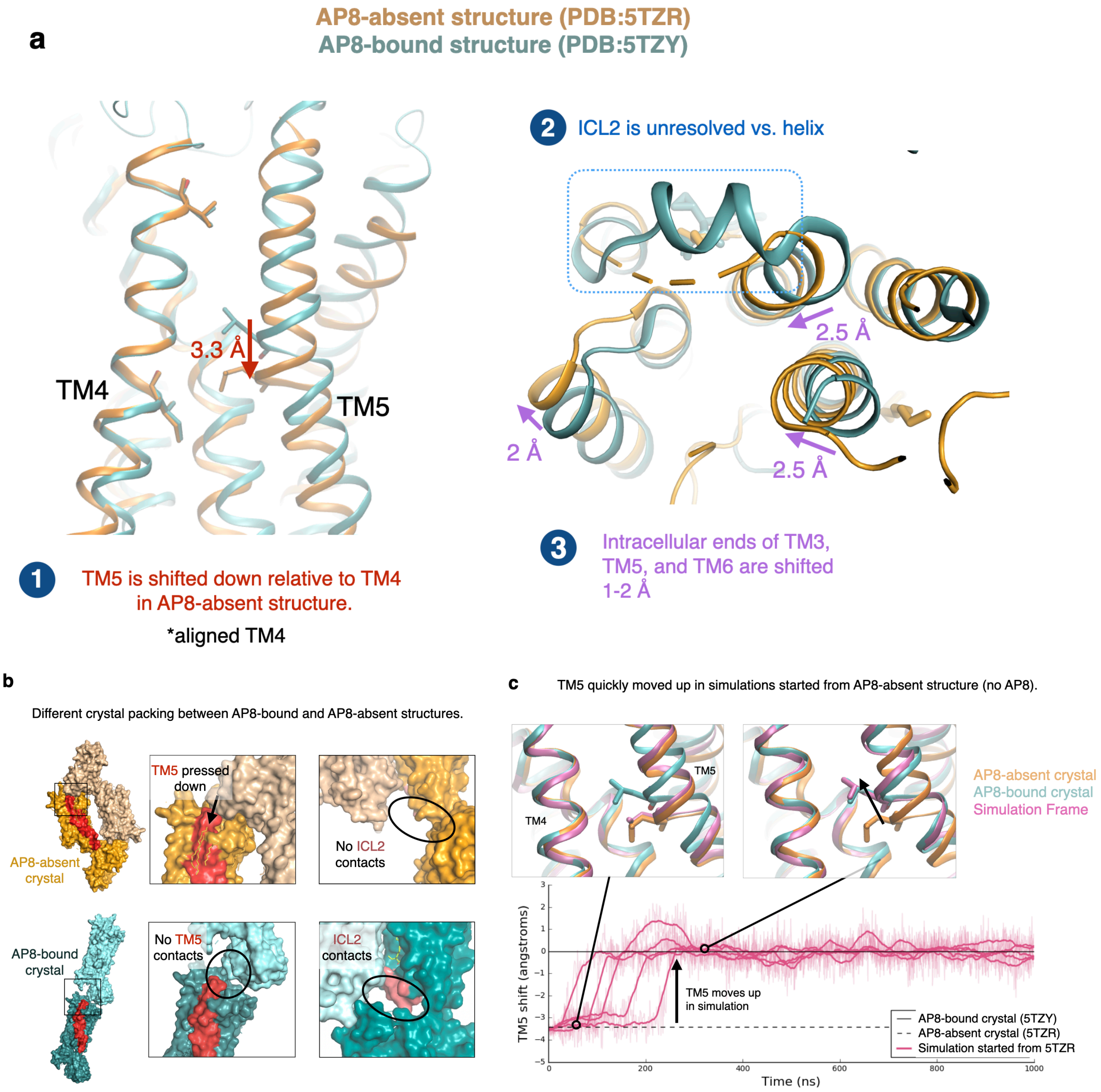

Supplementary Figure 2. Differences in receptor conformation in crystal structures may be a result of crystal packing, not ligand binding. (a) The conformation of TM5 differs in the AP8-bound and AP8-absent crystal structures, as pictured at left. The receptor is aligned to TM4 to show that TM5 is shifted 3.3 angstroms downward in the AP8-absent crystal structure. The intracellular ends of TM3, 5, and 6 are shifted by up to 2.5 angstroms between the AP8-bound and AP8-absent crystal structures, as pictured at right. ICL2 is helical in the AP8-bound structure and unresolved in the AP8-absent structure. The receptor was aligned to TM1, 2, 3, and 4. **(b)** The AP8-absent crystal structure has a different type of crystal packing than the AP8-bound structure, with neighboring subunits pressing TM5 (red) downward and less crystal packing around ICL2. **(c)** In simulations started from the AP8-absent crystal structure (no AP8 present), TM5 quickly shifts upward to the same conformation as in the AP8-bound crystal structure. This suggests the difference between the crystal structures is primarily due to the difference in crystal packing, rather than AP8 binding. Simulation trajectories show the TM5 shift for 5 independent simulations (see methods for details of the metric). Dashed line indicates the TM5 shift in the AP8-absent structure while the solid line is the TM5 shift in the AP8-bound crystal structure. Structures shown in snapshot images were aligned to TM4 of the AP8-bound crystal structure.

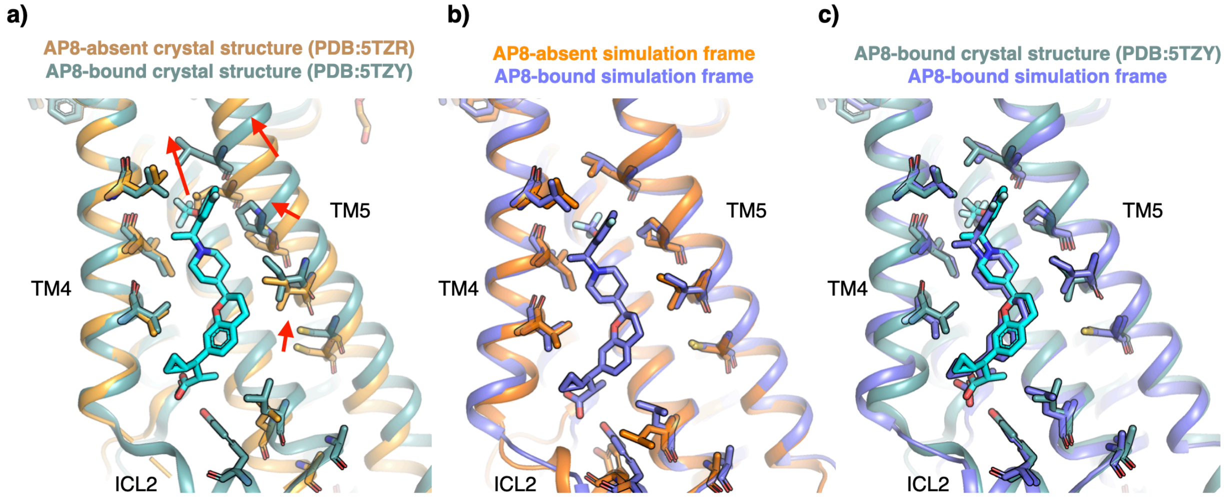

Supplementary Figure 3. The conformation of TM helices converges in simulations with and without AP8 bound. (a) Comparison of AP8-absent (yellow) and AP8-bound (green) crystal structures, showing significant differences in TM5 conformation around the AP8 binding site. **(b)** Representative frames from simulations of AP8-absent (orange) and AP8-bound (purple) systems, demonstrating convergence of TM helix conformations independent of the presence of AP8. The AP8-absent simulations were started from the AP8-absent crystal structure and the AP8-bound simulations from the AP8-bound crystal structure. Both frames shown are after 200 ns of simulation. **(c)** Overlay of AP8-bound crystal structure (green) and AP8-bound simulation frame (purple), illustrating that the conformation adopted in simulation matches the AP8-bound structure.

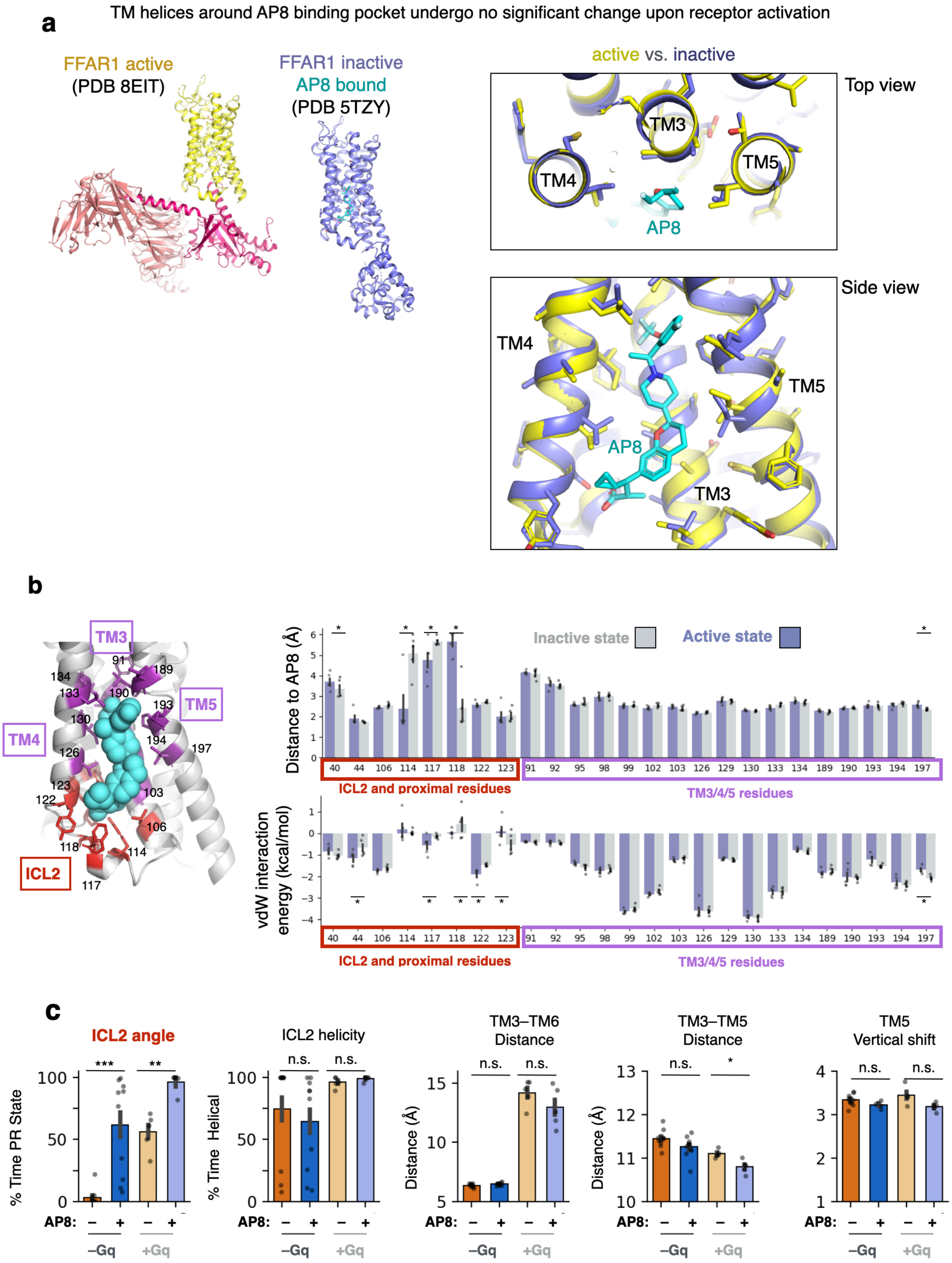

Supplementary Figure 4. Interactions between AP8 and TM helices are similar between active and inactive states of FFAR1. (a) Comparison of cryo-EM structure of the FFAR1-Gq complex (PDB 8EIT, yellow) with the

inactive, AP8-bound FFAR1 crystal structure (PDB 5TZY, purple). TM helices surrounding the AP8 binding site undergo little change upon receptor activation; the RMSD (root mean square deviation) of aligned residues shown in images is 0.65 Å (using residues 122-133, 94-106, and 190-201). **(b)** We used in-place docking (Schrodinger's Glide software) to calculate interaction metrics between AP8 and each residue in the binding site (within 4 Å of AP8 in the crystal structure). We used frames from two simulation conditions: the inactive WT receptor with AP8 bound (light grey) and the active G-protein complex with AP8 bound (blue) based on our model. Note that our active-state model is very similar to the recently published cryo-EM structure (PDB 8EIT) around the AP8 binding site. We used N=5 independent simulations per condition with 10 frames extracted from each simulation (every 100 ns) for analysis. The mean values for each metric and each residue were computed for the two conditions. The error bars show the 68% CI. Stars indicate which differences between interactions in active and inactive state are statistically significant (two-sided Mann Whitney U-test). For residues on the TM helices, only the difference in vdW interaction energy with residue 197 was statistically significant (*P=0.001*), but the interaction with AP8 actually became *less* favorable in the active state, and is therefore very unlikely to be a mechanism for agonism. **(c)** Comparing key metrics in simulations with and without AP8, and with and without Gq bound. Metrics shown correspond to those in Fig. 1; see Fig. 1 caption for full descriptions. *P*-values calculated using two-sided Mann Whitney U-test, from left *P*=0.0004, 0.007, 0.22, 0.58, 0.10, 0.22, 0.07, 0.01, 0.10, 0.098 with N=10 independent simulations for –Gq and N=5 independent simulations for +Gq.

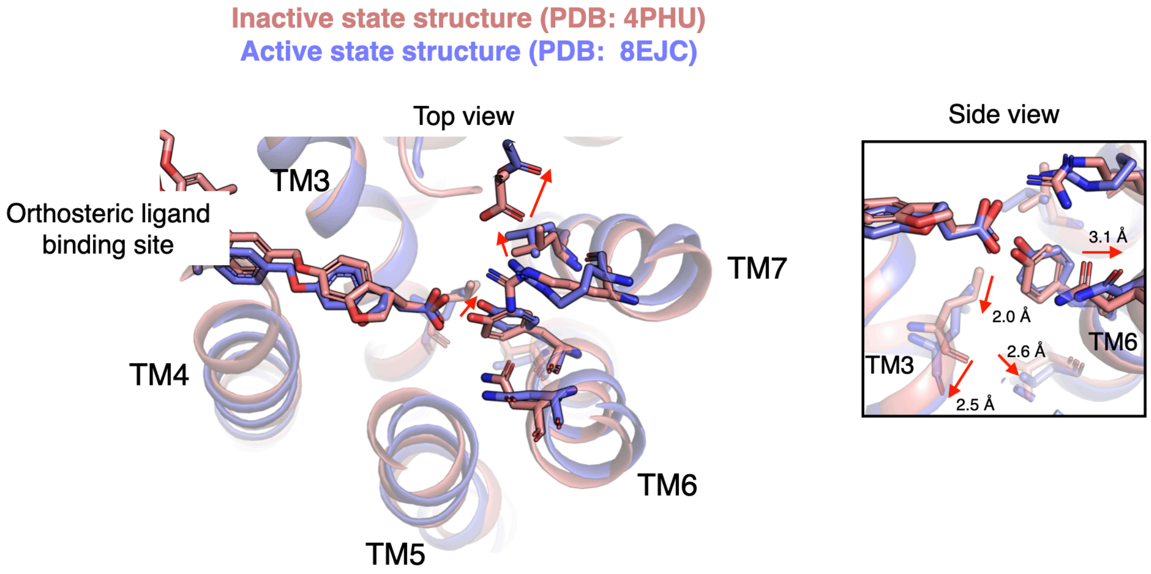

Supplementary Figure 5: Conformation of the orthosteric binding pocket differs between active and inactive structures. We observed rearrangements of several sidechains on TM3, TM6, and TM7, including changes in rotamers and hydrogen bonding interactions. To ensure consistency, we compared structures that both had the ligand TAK-875 bound. TAK-875 is an orthosteric partial agonist, very similar to MK-8666, that binds in the same site. There is presently no experimentally determined active state structure with MK-8666 bound.

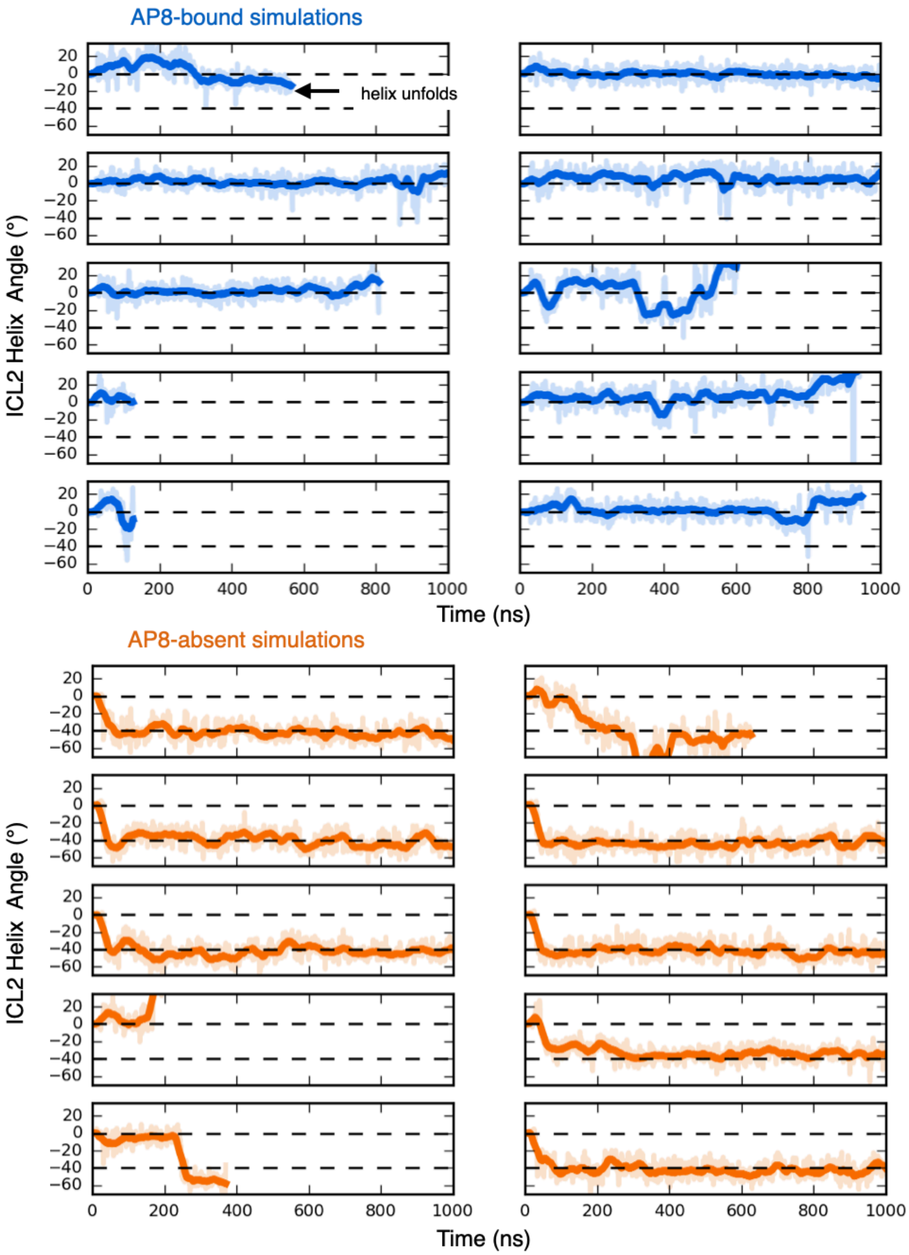

Supplementary Figure 6: AP8 modulates ICL2 angle in simulations. The ICL2 helix angle vs. time is shown for each independent simulation trial. The ICL2 angle was measured using the rotation of L112 and Y114 around the ICL2 helix axis for frames where ICL2 remained helical (see Methods). All simulations were the same length (2000 ns), though ICL2 helix unfolding could occur before that (trace ends early). For clarity only the first 1000 ns of the simulations are shown. The mean helix unfolding time is 1050 ns with AP8 bound and 3500 ns with AP8 absent, calculated by plotting the population of the folded state over time across the independent simulations for a condition, and fitting the curve to a decaying exponential to obtain the mean lifetime of the folded state. Angles corresponding to the two stable states are labeled with dashed lines. The top line, at 0 degrees, is the PR state. The bottom line, at –40 degrees, is the NR state. Simulations of the receptor with AP8 bound (blue) favor the PR state, whereas simulations without AP8 (orange) favor the NR state. All simulations were started from the crystal structure with AP8 bound; AP8 was removed in orange traces. Thick traces are a 15-ns moving average.

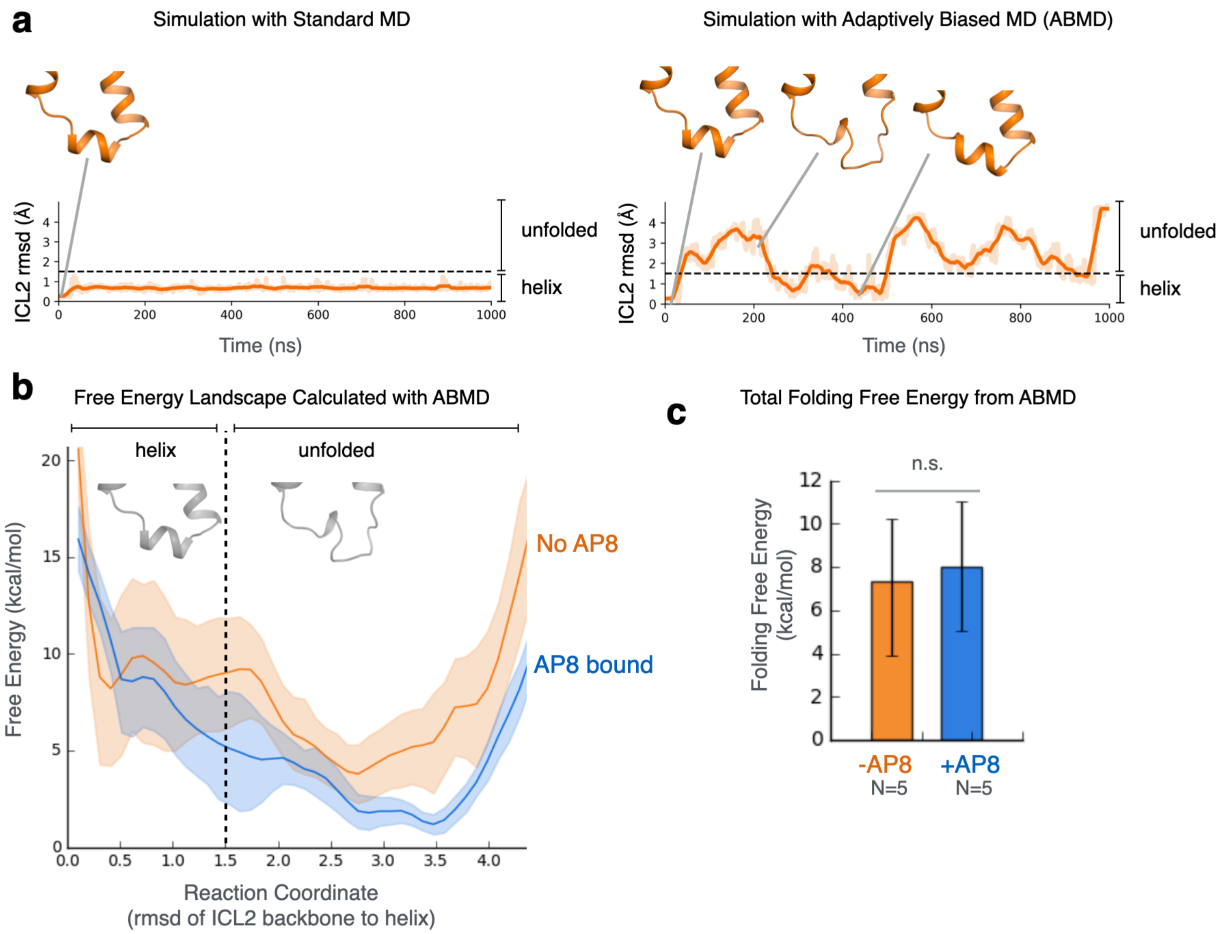

Supplementary Figure 7. Quantifying folding free energy of ICL2 helix with adaptively biased MD (ABMD). **(a)** Reaction coordinate chosen was the RMSD (root mean square deviation) of ICL2 backbone to the ICL2 helix. Using ABMD with this reaction coordinate led to more uniform sampling of the reaction coordinate and more frequent transitions between states as expected (right trace). This is in contrast to standard MD (left trace) which shows limited sampling of the conformational space. In the specific simulation shown at left, ICL2 remains helical, but in other simulations with no G protein present, it often unfolds and does not refold. Dashed lines indicates threshold between ICL2 helical state and unfolded state. **(b)** The free energy landscape across the helix folding reaction coordinate was calculated with and without AP8 bound. The results are the average of N=5 simulations for each condition. The shaded area shows the standard error of the mean. Dashed line indicates threshold between ICL2 helical state and unfolded state. The free energy profiles suggest that ICL2 favors an unfolded conformation in the absence of a bound G protein, whether or not AP8 is bound. While the AP8-bound crystal structure captures ICL2 in a folded helical conformation, there are several potential reasons for this discrepancy including specific crystal packing contacts (Supp. Fig. 2) and reduced (cryogenic) temperatures during crystallization that would favor a lower entropy state. **(c)** The net folding free energy was not significantly different with and without AP8 bound. To get the net folding free energy difference, we first calculated the relative probability of the folded vs. unfolded states by integrating the average free energy landscape from (b) using the partition function and defined threshold (dashed lines), and then transforming the relative probability to an energy difference. Error calculated from min and max curves of the energy landscape.

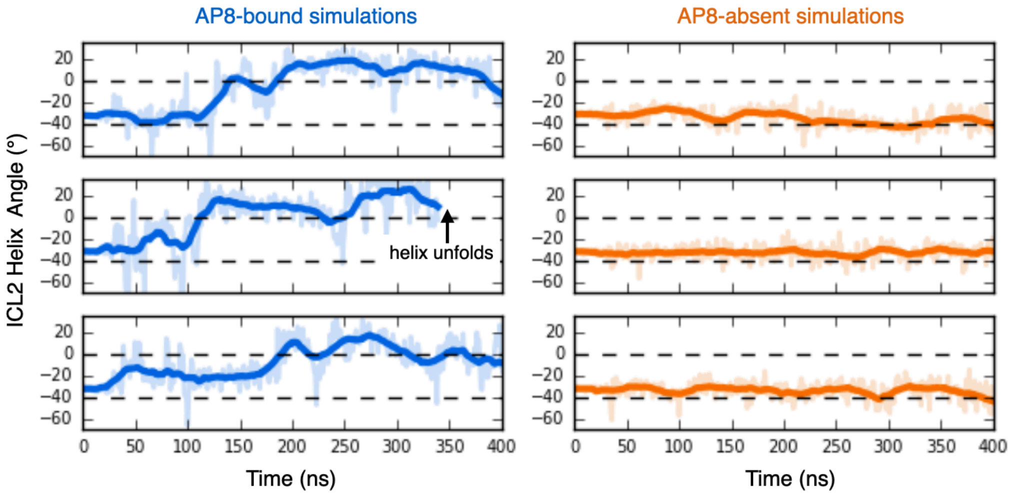

Supplementary Figure 8: When simulations are started from the rotated ICL2 state, with and without AP8 bound, we observe the same end result. The ICL2 angle vs. time is shown for each independent simulation trial. Thick traces are a 15 ns moving average. The ICL2 helix angle was measured using the rotation of L112 and Y114 around the ICL2 helix axis (see methods). Angles corresponding to the two stable states are labeled with dashed lines. The top line, at 0 degrees, is the PR state. The bottom line, at –40 degrees, is the NR state. Simulations of the receptor with AP8 bound (blue) favor the PR state while simulations without AP8 (orange) favor the NR state. Simulations were started from the NR state, which was modeled into the starting structure using Schrodinger (see methods). All simulations were the same length (2000 ns), though helix unfolding could occur before that (trace ends early).

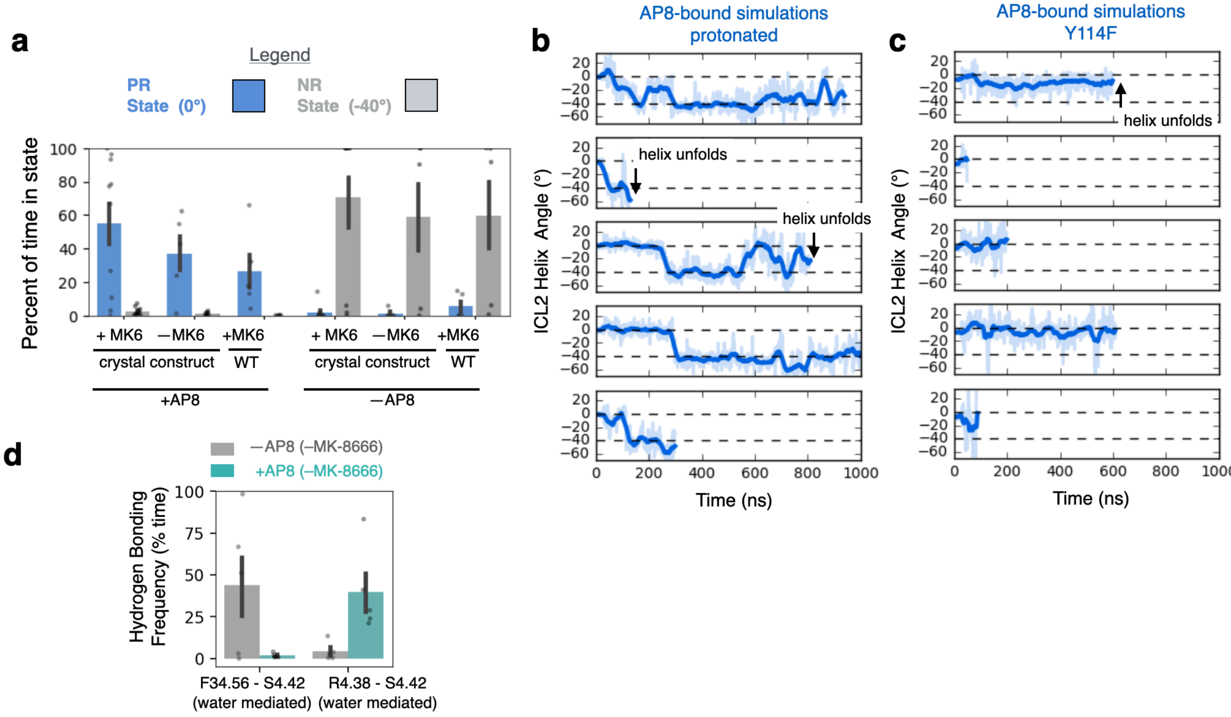

Supplementary Figure 9. Effect of AP8 on ICL2 across control and perturbed conditions. (a) Control simulations with partial agonist MK-8666 removed or engineered mutants reversed showed similar results. The major determinant

of ICL2 conformation was the presence or absence of AP8. Bars show the mean time spent in each ICL2 helix conformation, over the course of a 1 µs simulation for 6 unique conditions. Error bars show 68% CI, and from left to right, N=10, 5, 5, 10, 5, 5. **(b)** Disruption of key hydrogen bond negates AP8's effect on ICL2 conformation. The ICL2 angle vs. time is shown for each independent simulation. All simulations were the same length (1000 ns), though helix unfolding could occur before that (trace ends early). The top dashed line, at 0 degrees, is the PR state. The bottom dashed line, at –40 degrees, is the NR state. AP8's carboxylate group was protonated for the duration of these simulations, disrupting the water-mediated hydrogen bond to ICL2 backbone. This allowed the ICL2 helix to adopt the NR state, even with AP8 bound. **(c)** Similarly, Y114 was mutated to phenylalanine, disrupting the hydrogen bond between AP8 and the tyrosine hydroxyl. This hydrogen bond was less important for maintaining the PR state though resulted in faster ICL2 unfolding. All simulations were the same length (1000 ns), though helix unfolding could occur before that (trace ends early). **(d)** AP8 alters a network of polar interactions even in the absence of MK-8666; the frequency of water-mediated hydrogen bonds between key ICL2 residues were quantified in presence and absence of AP8 (and without MK-8666). Data presented as mean with 68% CI (N=5 independent simulations for each condition). Only simulation frames where ICL2 remained folded were used.

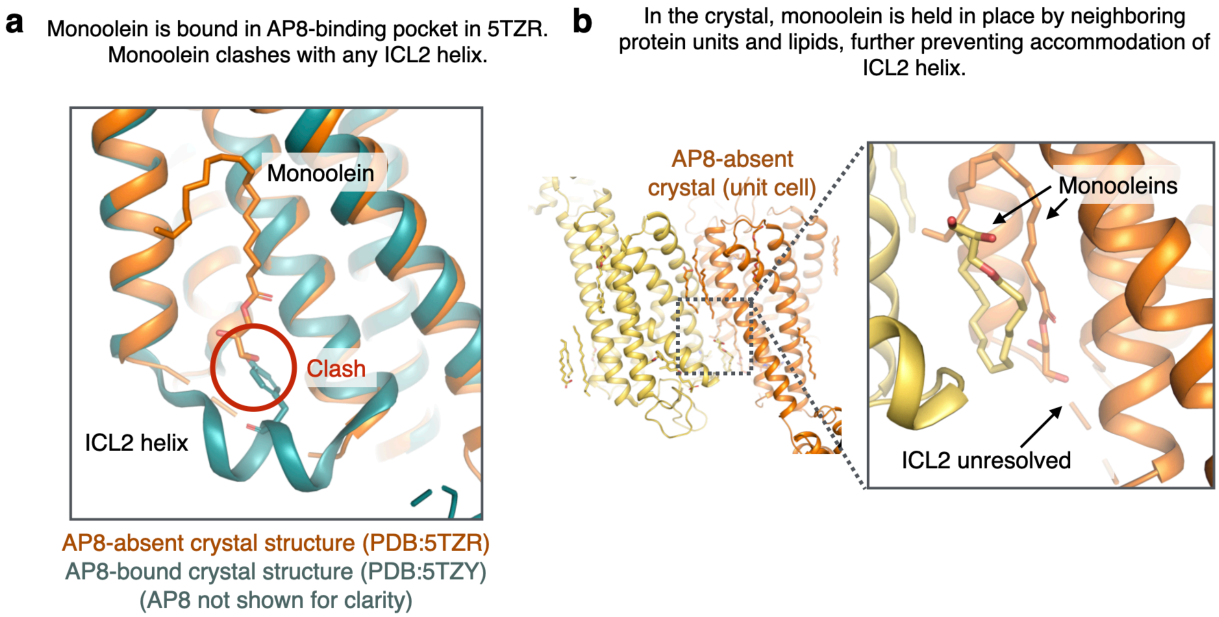

Supplementary Figure 10: A monoolein bound to AP8 site in AP8-absent crystal structure may perturb site. **(a)** In the AP8-absent crystal structure, ICL2 is unresolved, and we do not see the rotated helix conformation observed in simulation. A likely explanation for this discrepancy is that in the AP8-absent crystal structure, a monoolein lipid is bound to the ICL2 site, and would sterically clash with the ICL2 helix. The two crystal structures of FFAR1 are overlaid: the AP8-absent structure in orange and AP8-bound structure in green. AP8 is not shown for clarity. **(b)** The neighboring molecules in the crystal unit cell are shown. Monoolein is tightly packed at the AP8 site by contacts with neighboring lipid molecules and protein units.

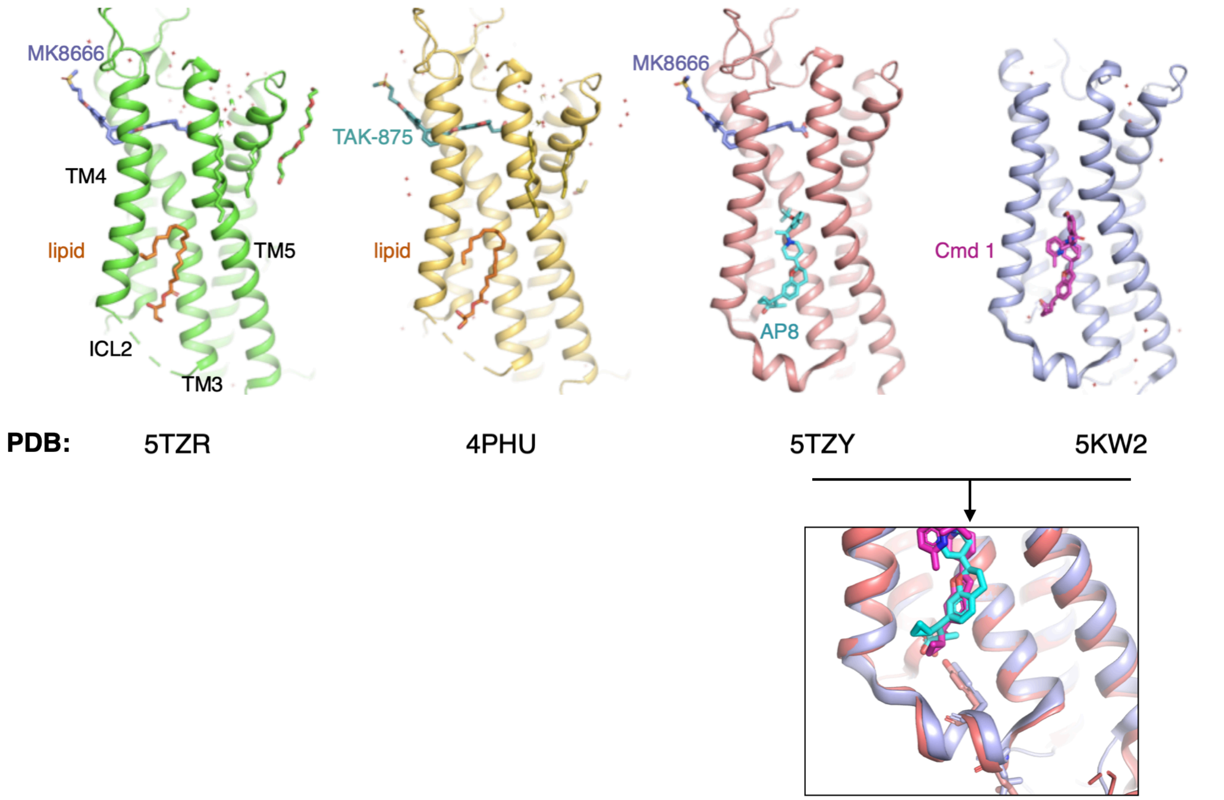

Supplementary Figure 11: Comparison of available crystal structures of FFAR1. Two structures (PDB: 5TZR and 4PHU) have only an orthosteric agonist bound. One structure (PDB: 5TZY) has both an orthosteric agonist and allosteric agonist bound. Another structure (PDB: 5KW2) has only an allosteric ligand (Cmd 1) bound, with no orthosteric ligand present. Notably, this AP8-like ligand (Cmd 1) interacts with ICL2 in the same way as AP8 does in the structure with MK-8666 (orthosteric agonist) present. In other words, AP8-like allosteric ligands stabilize ICL2 in the "PR" conformation, regardless of whether an orthosteric ligand is present or not.

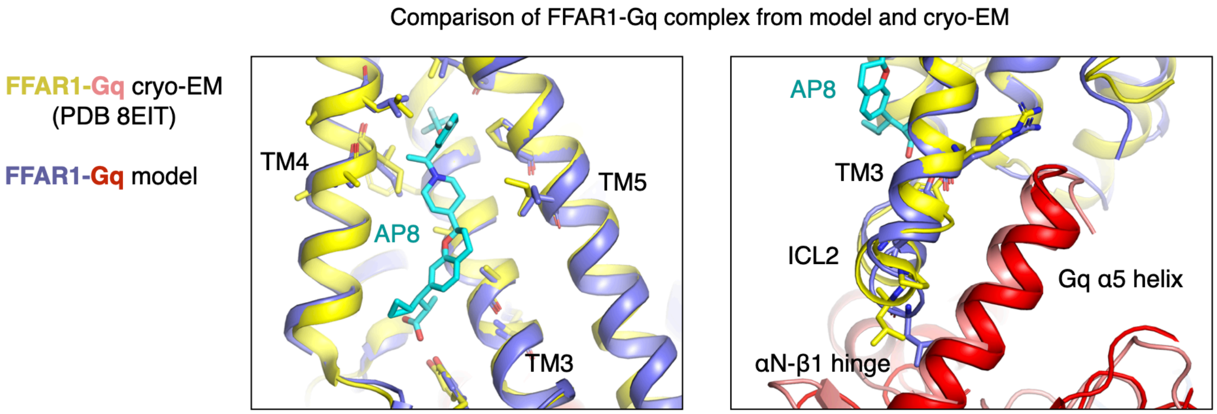

Supplementary Figure 12. Comparison of active-state model to cryo-EM of FFAR1-Gq. The AP8 binding pocket and the ICL2 interface in our model is very similar to that of the cryo-EM structure. The leucine on ICL2 engages the G protein (through the α5 helix and the αN-β1 hinge region) in both our model/simulations and in the cryo-EM structure.

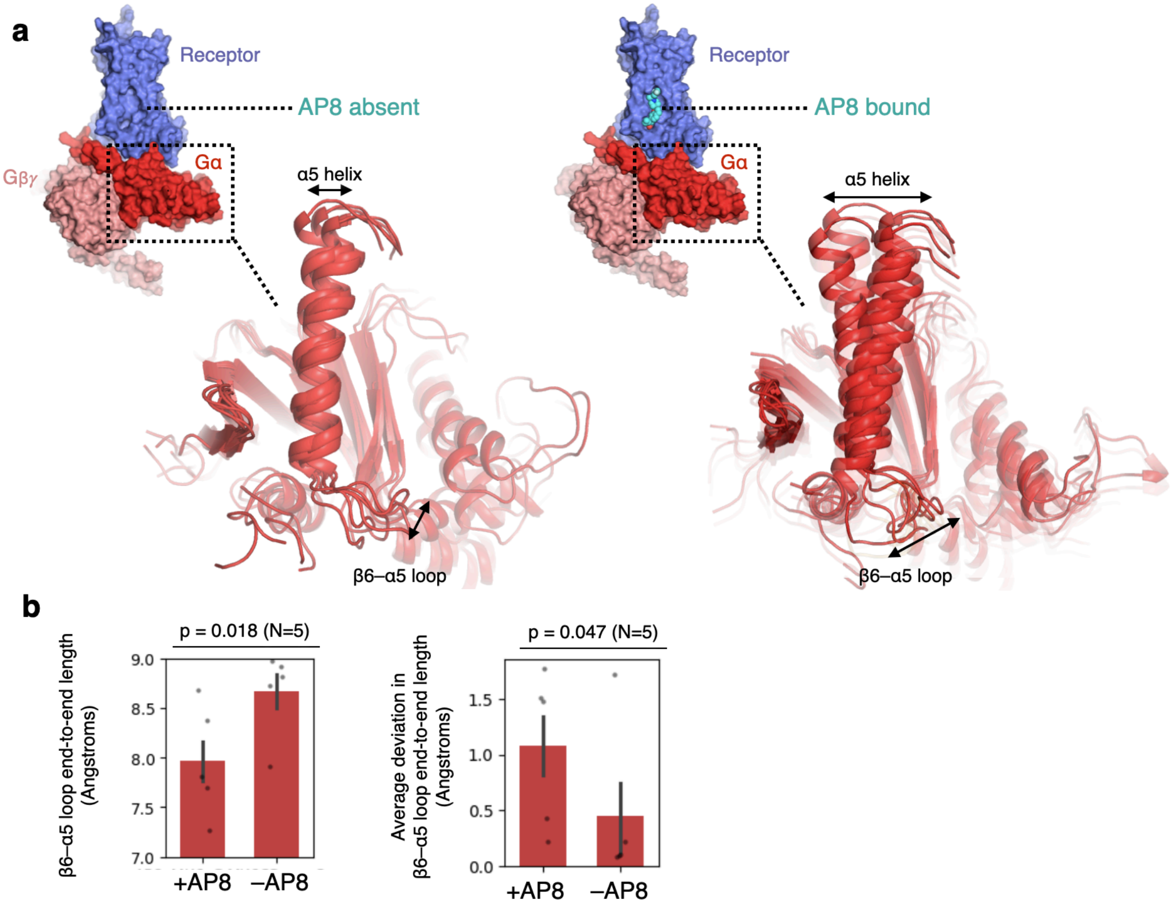

Supplementary Figure 13. G protein internal conformation and dynamics affected by the presence of AP8. (a) Cartoon shows an increase in dynamics of the α5 helix and connected β6-α5 loop in the presence of AP8. Five frames from different simulations, all taken at the 1000 ns timepoint, are overlaid for each condition. The Gα subunit is aligned to the stable β sheets to better visualize internal motions. **(b)** Comparison of conformation and dynamics with and without AP8 bound, using two metrics. The β6-α5 loop end-to-end length corresponds to length between residue 504 Cα to residue 508 Cα. Left: the end-to-end length of β6-α5 loop is slightly smaller with AP8. Right: the fluctuations in the end-to-end distance increased in the presence of AP8. Data presented as mean from 5 independent simulations for each condition, each 1 μs in length (error bars are 68% CI, *P*-values calculated using two-sided Mann-Whitney U test).

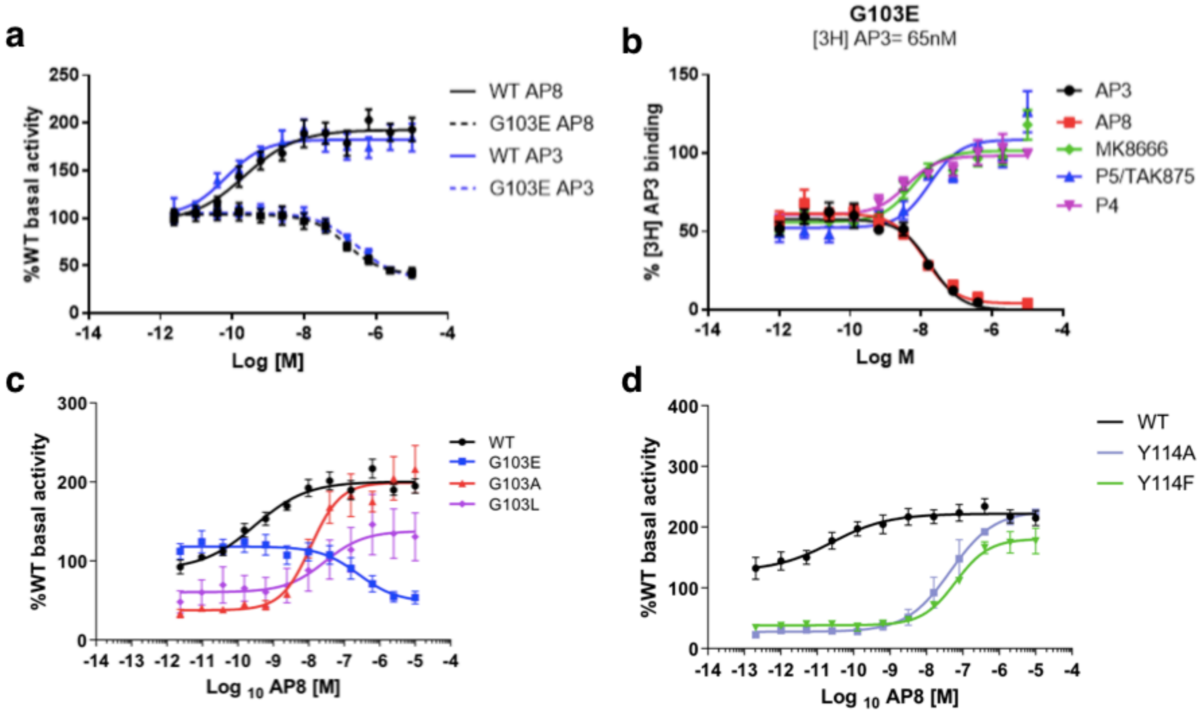

Supplementary Figure 14. Mutagenesis and binding experiments validate computational results. (a) G103E converts AP8 to an inverse agonist; consistent behavior was seen in AP3, an analogue of AP8, that binds at the same membrane-facing site[1]. GPR40 activity was monitored in IP1 accumulation assays in HEK293 cells expressing WT or mutant treated with AP8 or AP3. Data is plotted as the % of WT receptor basal activity (cells treated with 1% DMSO), where data points are mean ± S.E.M. from at least N=3 experiments. **(b)** Positive binding cooperativity between AgoPAM AP3 (a radiolabeled AP8-like ligand) and partial agonists MK-8666, TAK875, and P4, which all bind to the extracellular pocket, is maintained at the G103E receptor. The binding of radiolabeled AP3 was measured at varying concentrations of probe ligand (AP3, AP8, MK-8666, TAK875, and P4). Data points are mean ± S.E.M. from at least N=2 experiments. **(c, d)** Receptor activity was monitored in IP1 accumulation assays in HEK293 cells expressing WT or mutant treated with AP8. Each study was repeated N=2-5 times. **(c)** G103(3.49) mutants produced a range of AP8 efficacies. G3.49E had the lowest *in vitro* efficacy, G3.49L had an intermediate value, and finally G3.49A and WT(G3.49) had the highest values. **(d)** Mutant Y114A and Y114F disrupt the hydrogen bond interaction between Y114 and AP8, contributing to a reduced potency. Calculation of intrinsic efficacy[2,3] indeed showed a 10x decrease for AP8 (P=0.03, t-test), while the effect for MK-8666 was not statistically significant.

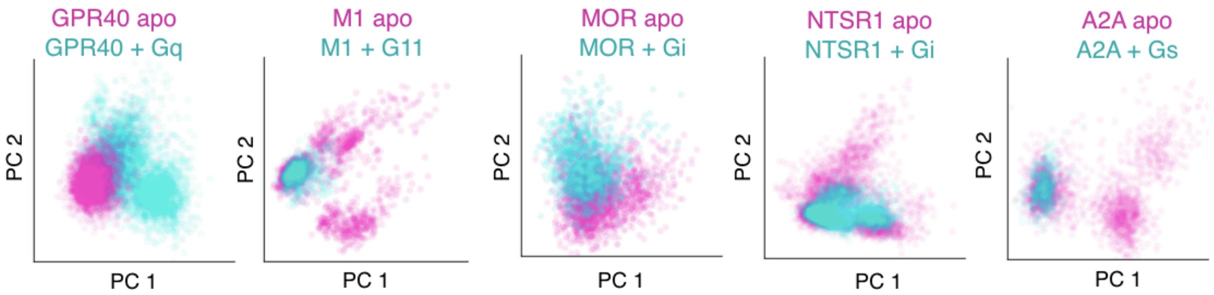

Supplementary Figure 15. Across diverse receptors, ICL2 helix conformation changes upon formation of receptor-G protein complex. Principal component analysis of ICL2 psi and phi angles using residues from 3.55 to 4.39 (Ballesteros-Weinstein numbering scheme). Cyan dots correspond to frames from simulations of receptor-G protein complex and pink dots are from receptor only simulations. Principal components were computed for each receptor independently.

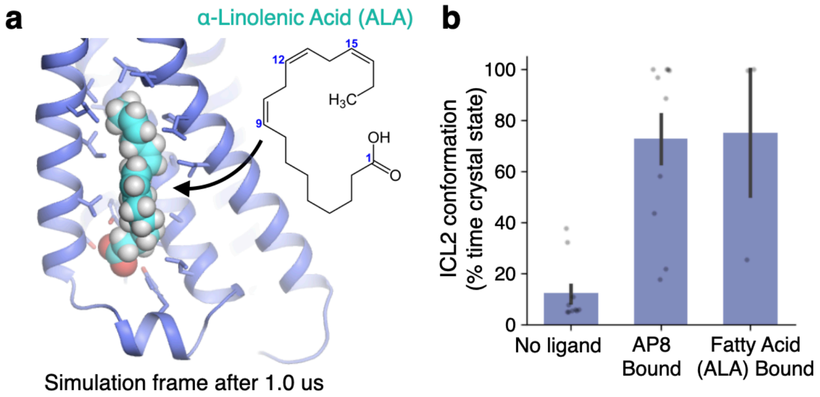

Supplementary Figure 16. Endogenous fatty acids also bind to and control membrane-facing ICL2 site. (a) Representative frame from simulation shows that α-linolenic acid (ALA) remains stably bound to the AP8-binding site for at least 1 us. **(b)** Fatty acids exert the same effect on ICL2 conformation as AP8. We measured the fraction of simulation time ICL2 spent in the crystal helix conformation. Data presented as mean with 68% CI (N=10, 10, and 5 independent simulations for unliganded, AP8-bound, and ALA-bound conditions, respectively).

Supplementary Table 1. Effect of mutations on basal activity and AP8 efficacy.

	Membrane Expression (% WT Expression)	Basal Activity (% WT Activity)	AP8 EC50/IC50 (nM)	AP8 Emax (% Basal WT Activity)
**WT**	100 (10)	105 ± 3 (3)	0.18 ± 0.14 (6)	213 ± 17 (6)
**G103E**	53 ± 5 (9)	103 ± 13 (3)	150 ± 13 (3)	42 ± 6 (3)
**G103A**	126 ± 18 (3)	37 ± 7 (2)	12 ± 1.3 (2)	200 ± 43 (2)
**G103L**	91 ± 6 (2)	63 ± 31 (2)	33 ± 6 (2)	136 ± 56 (2)
**G103D**	153 ± 16 (2)	12 ± 1 (3)	NA	NA
**Y114A**	86 ± 8 (3)	31 ± 5 (2)	70 ± 60 (2)	222 ± 12 (2)
**Y114F**	102 ± 11 (4)	38 ± 6 (2)	130 ± 48 (2)	184 ± 35 (2)
**A116G**	76 ± 4 (2)	53 (1)	0.05 ± 0.04 (2)	274 ± 10 (2)

*Data presented as mean ± S.E.M. (from N independent experiments). For each experiment, we performed n=2 technical replicates.

Supplementary Table 2. Effect of mutations on MK-8666 efficacy.

	EC50 (nM)	Emax (% Basal WT Activity)
**WT**	4.8 ± 1.9 (5)	202 ± 24 (4)
**G103E**	13 ± 3.6 (5)	121 ± 10 (4)
**G103A**	22 ± 2.9 (2)	152 ± 27 (2)
**G103D**	41 ± 16 (3)	127 ± 35 (3)
**G103L**	5.3 ± 2.2 (3)	92 ± 31 (3)
**Y114A**	5.0 ± 4.9 (2)	137 ± 13 (2)
**Y114F**	14 ± 0.1 (2)	133 ± 20 (2)
**A116G**	2.7 ± 2.3 (3)	189 ± 1 (3)

*Data presented as mean ± S.E.M. (N independent experiments). For each experiment, we performed n=2 technical replicates.

Supplementary Table 3. Binding Affinity of AP8 and analogs at WT and G103E construct

	Construct		
**Radioligand**	**GPR40 WT**	**G103E mutant**	**Y114F mutant**
[^{3}H]AP8	2.0 ± 0.5 (3)	28.5 ± 1.2 (3)	29.3 ± 0.5 (3)
[^{3}H]AP3	3.1 ± 1.8 (2)	55.6 ± 21.6 (2)	NA
[^{3}H]P4	0.9 ± 0.4 (3)	2.3 ± 0.3 (3)	1.6 ± 0.1 (3)

*Data presented as mean K_d (nanomolar) ± S.E.M. (N independent experiments). For each experiment, we performed n=2 technical replicates.

Supplementary Table 4. Simulation conditions

#	Name	Description	Trials	Length (µs)
1	AP8-bound	Receptor structure from PDB:5TZY with bound ligands (AP8 and MK-8666)	10	2
2	AP8-absent	Receptor structure from PDB:5TZY, AP8 removed, MK-8666 bound	10	2
3	AP8-bound, MK8-absent	Receptor structure from 5TZY, MK-8666 removed, AP8-bound	10	2
4	AP8-absent, MK8-absent	Receptor structure from PDB:5TZY, AP8 removed, MK-8666 removed	10	2
5	AP8p-bound	Receptor structure from PDB:5TZY, AP8 protonated	5	1
6	AP8-bound, start rotated	Same setup as 1, but ICL2 modeled to start at rotated state (modeled from condition 2 simulation)	5	1
7	AP8-absent, start rotated	Same setup as 2, but ICL2 modeled to start at rotated state (modeled from condition 2 simulation)	5	1
8	AP8-bound ABMD	Same setup as 1, but using adaptively biased MD with reaction coordinate as ICL2 helicity	5	1
9	AP8-absent ABMD	Same setup as 2, but using adaptively biased MD with reaction coordinate as ICL2 helicity	5	1
10	AP8-bound G103E	Same setup as 1, G103E mutant modeled	10	2
11	AP8-absent G103E	Same setup as 2, G103E mutant modeled	10	2
12	AP8-bound, MK8-absent G103E	Same setup as 3, G103E mutant modeled	10	2
13	AP8-absent, MK8-absent G103E	Same setup as 4, G103E mutant modeled	10	2
14	AP8-bound, Y114F	Same setup as 1, Y114F mutant modeled	5	1
15	AP8-bound, G103	Same setup as 1, G103 to replace A103 in crystal construct	5	1
16	AP8-bound, G103L	Same setup as 1, G103L mutant modeled	5	1
17	AP8-absent 5TZR	Receptor structure from 5TZR (AP8-absent crystal structure)	5	1
18	AP8-bound, Gq-bound	Active state model of FFAR1 with bound-AP8 and heterotrimeric Gq	5	1
19	AP8-absent, Gq-bound	Active state model of FFAR1 with bound-AP8 and heterotrimeric Gq	5	1
20	AP8-bound, Gq-bound, G103E	Same as 18, G103E mutant modeled	5	1
21	AP8-absent, Gq-bound, G103E	Same as 19, G103E mutant modeled	5	1
22	MOR receptor, Gi-bound	Receptor and G-protein complex from PDB: 6DDF	5	1
23	MOR receptor	Same structure as 22, but G protein removed	5	1
24	M1 receptor, G11-bound	Receptor and G-protein complex from PDB: 6OIJ	5	1
25	M1 receptor	Same structure as 24, but G protein removed	5	1
26	NTSR1 receptor, Gi-bound	Receptor and G-protein complex from PDB: 6OS9	5	1
27	NTSR1 receptor	Same structure as 26, but G protein removed	5	1
28	A2A receptor, Gs-bound	Receptor and G-protein complex from PDB: 6GDG	5	1
29	A2A receptor	Same structure as 28, but G protein removed	5	1

Supplementary Table 5. Simulations Setup Details

**Receptor-only Simulations**	
Box Dimensions	80 Å x 90 Å x 85 Å
Salt Concentration	150 mM sodium chloride
Total Atoms	62,000
Total Waters	13,300
Total Lipids	130
Lipid Type	palmitoyl-oleoyl-phosphatidylcholine (POPC)
**Receptor-Gq Complex Simulations**	
Box Dimensions	120 Å x 120 Å x 140 Å
Salt Concentration	150 mM sodium chloride
Total Atoms	98,000
Total Waters	21,000
Total Lipids	180
Lipid Type	palmitoyl-oleoyl-phosphatidylcholine (POPC)

*Data are approximate values for box dimensions, total atoms, total waters, and total lipids.

Supplementary References

1. Pachanski, M. J. *et al.* GPR40 partial agonists and AgoPAMs: Differentiating effects on glucose and hormonal secretions in the rodent. *PLOS ONE* **12**, e0186033 (2017).
2. Black, J. W. & Leff, P. Operational models of pharmacological agonism. *Proceedings of the Royal Society of London. Series B. Biological Sciences* **220**, 141–162 (1983).
3. Kenakin, T., Watson, C., Muniz-Medina, V., Christopoulos, A. & Novick, S. A simple method for quantifying functional selectivity and agonist bias. *ACS Chemical Neuroscience* **3**, 193–203 (2012).
